# Identification of a Novel Biosynthetic Gene Cluster in *Aspergillus niger* Using Comparative Genomics

**DOI:** 10.3390/jof7050374

**Published:** 2021-05-11

**Authors:** Gregory Evdokias, Cameron Semper, Montserrat Mora-Ochomogo, Marcos Di Falco, Thi Truc Minh Nguyen, Alexei Savchenko, Adrian Tsang, Isabelle Benoit-Gelber

**Affiliations:** 1 Centre for Structural and Functional Genomics, Department of Biology, Concordia University, 7141 Rue Sherbrooke Ouest, Montréal, QC H4B 1R6, Canada; gregory.evdokias@gmail.com (G.E.); montse.mora.ochomogo@gmail.com (M.M.-O.); marcos.difalco@concordia.ca (M.D.F.); thitrucminh.nguyen@concordia.ca (T.T.M.N.); adrian.tsang@concordia.ca (A.T.); 2 Department of Microbiology, Immunology and Infectious Disease, University of Calgary, 3330 Hospital Drive, Calgary, AB T2N 4N1, Canada; cjsemper@ucalgary.ca (C.S.); alexei.savchenko@ucalgary.ca (A.S.)

**Keywords:** *Aspergillus niger*, secondary metabolites, BGC: biosynthetic gene cluster, NRPS: nonribosomal peptide synthetase, comparative genomics, CRISPR/Cas9

## Abstract

Previously, DNA microarrays analysis showed that, in co-culture with *Bacillus subtilis*, a biosynthetic gene cluster anchored with a nonribosomal peptides synthetase of *Aspergillus niger* is downregulated. Based on phylogenetic and synteny analyses, we show here that this gene cluster, *NRRL3_00036-NRRL3_00042*, comprises genes predicted to encode a nonribosomal peptides synthetase, a FAD-binding domain-containing protein, an uncharacterized protein, a transporter, a cytochrome P450 protein, a NAD(P)-binding domain-containing protein and a transcription factor. We overexpressed the in-cluster transcription factor gene *NRRL3_00042*. The overexpression strain, NRRL3_00042^OE^, displays reduced growth rate and production of a yellow pigment, which by mass spectrometric analysis corresponds to two compounds with masses of 409.1384 and 425.1331. We deleted the gene encoding the NRRL3_00036 nonribosomal peptides synthetase in the NRRL3_00042^OE^ strain. The resulting strain reverted to the wild-type phenotype. These results suggest that the biosynthetic gene cluster anchored by the *NRRL3_00036* nonribosomal peptides synthetase gene is regulated by the in-cluster transcriptional regulator gene *NRRL3_00042*, and that it is involved in the production of two previously uncharacterized compounds.

## 1. Introduction

The filamentous fungus *Aspergillus niger* has long been established as an industrial microorganism for the production of enzymes and organic acids. *Aspergillus niger* is capable of high secretion of proteins such as glucoamylase [1] and organic acids such as citric, gluconic and oxalic acids [2]. *Aspergillus niger* has a versatile lifestyle and can be isolated from many ecological niches, including soil and decaying plant materials, where it encounters other microorganisms. It can be found as a food contaminant [3], a plant parasite and endophyte [4]. The ability of *A. niger* to thrive in diverse environments is correlated with its capacity to produce a wide repertoire of secondary metabolites [5]. It was previously reported that *A. niger* and *Bacillus subtilis* altered their metabolism when cultivated together. The whole transcriptome of *A. niger* during its interaction with *B. subtilis* in co-cultures was analyzed by DNA microarrays. Among other changes, a predicted biosynthetic gene cluster (BGC) in *A. niger* was down-regulated in the co-cultures compared to the mono-cultures [6]. Using the functional annotation of *A. niger* NRRL3 [7], this predicted biosynthetic gene cluster harbors genes encoding: a nonribosomal peptide synthetase (NRPS) (NRRL3_00036), a transporter (NRRL3_00039), a cytochrome P450 (NRRL3_00040), a FAD-binding domain-containing protein (NRRL3_00037) and a NAD(P)-binding domain-containing protein (NRRL3_00041). To our knowledge, this BGC has not been described previously. Nonribosomal peptides, products of NRPS, are of interest due to their diverse bioactivities such as toxins, siderophores, pigments, antibiotics, cytostatics, immunosuppressants or anticancer agents [8]. Eighteen NRPS have been found is the genome of *A. niger*. In this study, we describe the overexpression of the transcription factor gene *NRRL3_00042* of a novel BGC, anchored by the NRPS gene *NRRL3_00036*, and the resulting overproduction of new secondary metabolites.

## 2. Materials and Methods

Strains and culture conditions. The strain *A. niger* CSFG_7003 (NRRL2270 *∆pyrG ∆kusA*) was used as parental strain for the overexpression of the gene encoding the transcription factor NRRL3_00042. The NRRL3_00042 overexpressing strain was generated by replacement of the glucoamylase gene using the CRISPR/Cas9 genome editing method [9]. The *NRRL3_00042* gene was amplified by PCR and inserted into a plasmid containing 655 and 700 bp of the glucoamylase promoter and the terminator regions, respectively. The resulting plasmid together with a CRISPR/Cas9 plasmid containing a gRNA targeting the glucoamylase gene were co-introduced into CSFG_7003. The *A. niger NRRL3_00042* overexpressing strain (NRRL3_00042^OE^) was used as the host strain for the deletion of the NRPS gene *NRRL3_00036*, using the CRISPR/Cas9 genome editing method [9]. The primers, the rescue oligonucleotide for *NRRL3_00036* deletion and the genetic information of the strains used in this study are listed in Tables S1 and S2, respectively. The expression of the genes *NRRL3_00036* and *NRRL3_00042* in the NRRL3_00042^OE^ and CSFG_7003 strains was verified by RT-PCR. The β-tubulin gene was chosen as positive control. Total RNA was extracted from the NRRL3_00042^OE^ and CSFG_7003 strains using TRIzol reagent and treated with amplification-grade DNase I (Invitrogen). Complementary DNA (cDNA) was synthesized with the Improm-II reverse transcription kit (Promega) using the oligo-dT primer according to the manufacturer’s protocol. The cDNA was amplified using Phusion DNA polymerase (New England Biolabs, NEBS, Ipswich, MA 01938, United States) using the primers listed in Table S1, with annealing occurring at 64 °C and extension at 72 °C per the manufacturer’s recommendation.

*Aspergillus niger* gene transformation. Fungal spores at a final concentration of 5 × 10^6^ spores/mL were inoculated in 250 mL of liquid minimal medium “J” [10] with 10 mM uridine. Protoplasts were prepared by incubating mycelium for three hours at 37 °C in digestion solution [40 mg/mL VinoTaste Pro (Novozymes, A/S, Krogshøjvej 36, 2880 Bagsvaerd, Denmark), 1.33 M sorbitol, 20 mM MES pH 5.8, 50 mM CaCl_2_]. PEG-mediated transformation was performed as described in [9]. Three colonies from each transformation plate were isolated and purified on *Aspergillus* minimal medium with 1% maltose. To confirm successful gene replacement, the *gla*A locus of the purified transformants was amplified by PCR and profiled by restriction enzyme digestion (Figure S1).

Sample preparation for liquid chromatography mass spectrometry. Liquid stationary cultures were performed in 96-well plates containing *Aspergillus* minimum medium with 1% maltose, incubated during 5 and 12 days at 30 °C. From the stationary cultures, 75 µL of culture media were collected in 1.5 mL microfuge tubes and centrifuged at 16,000× *g* for 45 min to remove mycelia. The supernatants were transferred to new tubes and two volumes of cold methanol (−20 °C) were added for protein precipitation. Following incubation on ice for 10 min, samples were centrifuged at 16,000× *g* for 45 min to remove the precipitated proteins. Supernatants were transferred to fresh tubes and an equal volume of 0.1% formic acid was added. Methanol extracted metabolites were stored at −80 °C until LC-MS analysis was performed.

High-performance liquid chromatography-mass spectrometry (HPLC-MS) analysis of metabolites. Ten µL of each sample were injected into a Kinetex 150 × 2.1 mm, 5 µm, C18 column (Phenomenex, Torrence, CA, USA) for gradient separation of components using an Agilent 1260 Infinity II HPLC system (Agilent technologies, Santa Clara, CA, USA). The solvents used to generate the gradient during reversed-phase separation were 0.1% formic acid in water for Solvent A and 0.1% formic acid in acetonitrile for Solvent B. Solvent flow rate was 250 µL/min and the gradient conditions were 3% B isocratic for 1 min, increased to 80% B over 10 min, increased to 95% B in 0.1 min, maintained at 95% for 1 min, decreased to 3% B in 0.1 min and kept at 3% B for 4.8 min. Column eluate was delivered to a 7-Tesla Finnigan LTQ-FT mass spectrometer (Thermo Electron Corporation, San Jose, CA, USA) for electrospray ionization-MS. Ionization voltage used was 4900 V in positive mode and 3700 V in negative mode. Scan range was from 100 to 1400 *m*/*z* at 100,000 resolution at 200 *m*/*z*.

HPLC-MS data analysis. Differential compound production analysis was carried out using extracted chromatogram peak areas determined by Compound Discoverer 3.1 software (Thermo-Fisher Scientific, Waltham, MA, USA). The criteria used for compound detection and ion chromatogram extraction were 7 ppm for mass accuracy and a 0.2 min retention time window. Compound annotation was carried out using a 5 ppm mass accuracy matching against both a database of 968 *Aspergillus* associated metabolites put together from literature searches as well as the chemical entities of biological importance (ChEBI) database and the MassBank database available within ChemSpider [11].

Antimicrobial assay. Bacterial strains *Escherichia coli* JW5503–1 and *Staphylococcus aureus* N315 were grown in Mueller-Hinton II broth (bivalent cation adjusted) and prepared using the microdilution method according to CLSI parameters [12]. Metabolites were extracted using methanol, filtered, and dried using a Speedvac centrifuge. The dried extracts were weighed and re-suspended in DMSO at a concentration of 50 mg/mL. Extracts were added to bacterial cultures to a final concentration of 2.5 mg/mL and growth at 37 °C was monitored over a 20-h period by measuring OD600 at 30-min intervals. The antibiotic gentamicin (0.75 μg/mL) was used as positive control. The negative control was DMSO only. Three biological replicates were performed.

Phylogenetic tree construction and synteny. Protein sequences of 737 published fungal genomes were downloaded from the JGI MycoCosm database [13] on December 10, 2020. For each species, only one representative genome was selected. After removing the redundancy, there were 644 fungal genomes included in the analysis, which cover the 28 clades of the JGI MycoCosm taxonomic tree. The orthologs of the NRRL3_00036 protein were identified by performing the BLASTP search [14] against the collected fungal proteins with the e-value not greater than 1E-10 and the percent sequence identity and the query coverage at least 40% and 70%, respectively. Full-length protein sequences of the orthologs was aligned by using MUSCLE [15]. The multiple sequence alignment profile was then input into FastTree [16] to build the phylogenetic tree using the maximum-likelihood algorithm. The tree was finally edited by the iTOL program [17]. The synteny analysis was first conducted using FungiDB [18] and manually expended using the JGI MycoCosm database [13].

## 3. Results

### 3.1. Biosynthetic Gene Cluster Structure and Phylogeny

**Figure 1:**
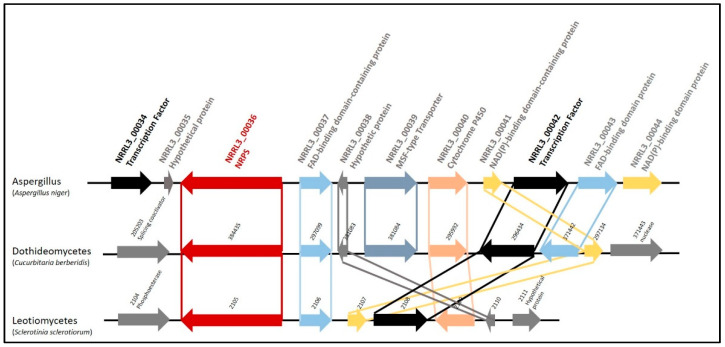
*NRRL3_00036* biosynthetic gene cluster structure and synteny. Shown are organizations of the *NRRL3_00036* cluster of *Aspergillus* (represented by *A. niger*), Dithidiomycetes (represented by *Cucurbitaria berberidis*) and Letiomycetes (represented by *Sclerotinia sclerotiorum*).

Using the functional annotation of *A. niger* NRRL3, the predicted biosynthetic gene cluster structure was identified as the following, a NRPS *(NRRL3_00036*), a transporter (*NRRL3_00039*), a cytochrome P450 (*NRRL3_00040*), a FAD-binding domain-containing protein (*NRRL3_00037*) and a NAD(P)-binding domain-containing protein (*NRRL3_00041*). In addition to the BGC, two genes encoding transcription factors, *NRRL3_00034* and *NRRL3_00042*, were found upstream and near the downstream boundary of the BGC (Figure 1). We conducted synteny analysis and found syntenic BGCs in *Aspergillus* species belonging to the Nigri and Candidi sections as well as species in the Dithidiomycetes and Letiomycetes. The organization of the cluster is different for the three groups (Figure 1). The functional annotation of the BGC anchored by *NRRL3_00036* revealed two co-localized genes encoding Zn2Cys6 transcription factors, *NRRL3_00034* and *NRRL3_00042*. However, the synteny analysis shows that only orthologs of *NRRL3_00042* were found in syntenic BGCs of the Dithidiomycetes and Letiomycetes. Furthermore, the downregulation of genes observed in the *A. niger* and *Bacillus subtilis* co-culture extends from *NRRL3_00035* to *NRRL3_00043* [6]. Taken together, these findings suggest that *NRRL3_00042* is likely to be involved in the regulation of the *NRRL3_00036* cluster and that the transcription factor *NRRL3_00034* and the NAD(P)-binding domain protein gene *NRRL3_00044* are not part of the cluster (Figure 1).

**Figure 2:**
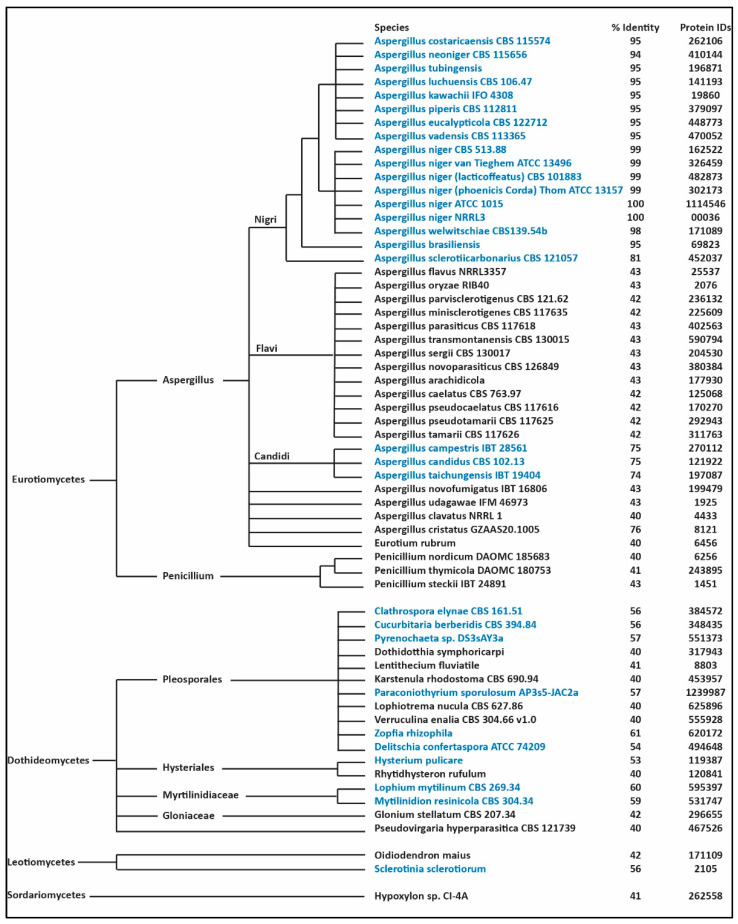
Phylogenetic tree built from orthologs of NRRL3_00036. Highlighted in blue are the syntenic species. The protein identification numbers (IDs) refer to the NRPS orthologs as assigned in the JGI MycoCosm database [13].

To get further insights on the biological function of the NRPS NRRL3_00036 and its biosynthetic gene cluster, we examined 737 sequenced, annotated and publicly available fungal genomes that represent the diversity of species in terms of evolutionary distance. Using BLASTP, we identified 58 orthologous genes of the NRPS NRRL3_00036, all were found within the Eurotiomycetes, the Dothidiomycetes and Letiomycetes (Figure 2). The analysis showed that species containing orthologs with sequence identity > 50% compared to NRRL3_00036 also harbor BGCs in synteny with the *NRRL3_00036* BGC. In one species, *Aspergillus cristatus*, the NRRL3_00036 orthologue has a sequence identity of 76% but the cluster is truncated with genes encoding the cytochrome P450 and the transporter missing. These two missing genes are not found elsewhere in the genome based on BLASTP analysis.

### 3.2. Overexpression of the In-Cluster Transcription Factor Gene Results in Impaired Growth and Overproduction of New Compounds

**Figure 3:**
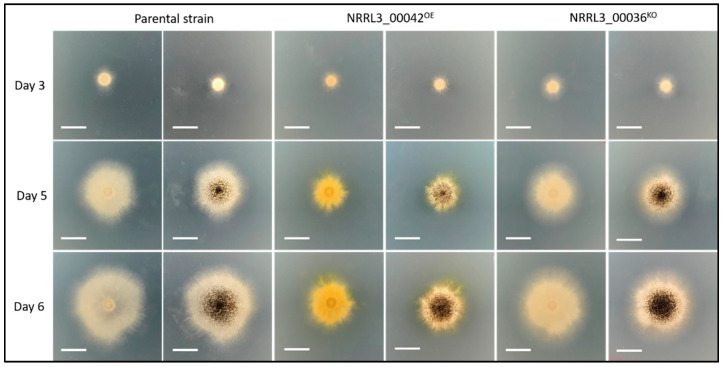
Growth profile of strains CSFG_7003 (parent), NRRL3_00042^OE^ and NRRL3_00042^OE^ _ΔNRRL3_00036 on agar plates containing minimum medium with 1% maltose. The scale bar represents 1 cm. Shown are images taken from the bottom (left) and the top (right) of the plates.

We overexpressed the gene *NRRL3_00042*, encoding a transcription factor located in the BGC anchored by *NRRL3_00036*. We inserted the open-reading frame of *NRRL3_00042* between the promoter and terminator of the glucoamylase gene by gene replacement, resulting in the strain named NRRL3_00042^OE^. The correct insertion was checked by PCR (Figure S1). To determine the expression of the transcription factor gene *NRRL3_00042* and the NRPS gene *NRRL3_00036*, we performed a RT-PCR on the parental strain CSFG_7003 and the NRRL3_00042^OE^ strain under the same growth conditions (Figure S2). We amplified a 990 bp and a 794 bp segment respectively as well as a 500 bp segment of *β-tubulin* as expression control [19]. The results demonstrated the overexpression of the transcription factor and the NRPS gene *NRRL3_00036* in the NRRL3_00042^OE^ strain compared to the expression level in the parental strain. The parental strain and the mutant strains were inoculated by 2 µL at 2000 spores/µL on agar minimum medium plates with 1% maltose as inducer. The comparative growth profile showed secretion of yellow pigments and impaired growth for the NRRL3_00042^OE^ strain (Figure 3). We had examined a second independent NRRL3_00042^OE^ strain which also displayed impaired growth and secretion of yellow pigments.

**Figure 4:**
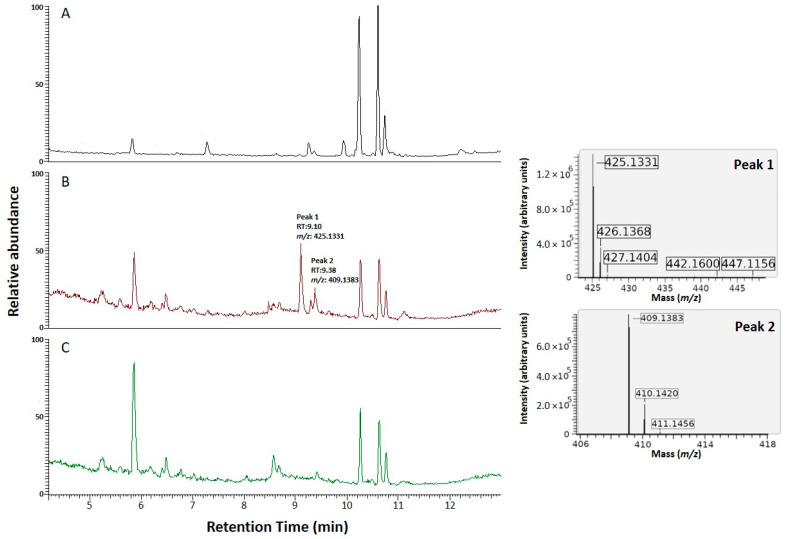
HPLC-MS analysis of extracts from 6-days-old MM 1% maltose cultures of *A. niger* strains. (**A**) Total ion chromatogram (TIC) of the parent strain CSFG_7003; (**B**) TIC of the mutant NRRL3_00042^OE^ strain, corresponding masses and adducts under peak 1 and peak 2 are shown on the right; (**C**) TIC of the mutant NRRL3_00042^OE^ _ΔNRRL3_00036 strain.

The promoter of the glucoamylase is inducible by the presence of maltose. The NRRL3_00042^OE^ strain was grown in stationary cultures containing 1% maltose for overexpression of the transcription factor (Figure S2). Five days after inoculation, the secreted metabolites were extracted and analyzed by HPLC-MS. The metabolic profile of strain NRRL3_00042^OE^ showed distinct and unique features compared to the parental strain (Figure 4). Analysis of the total ion chromatogram (TIC) revealed two peaks (RT = 9.10, RT = 9.38), corresponding to two compounds with respective masses of 425.1331 and 409.1384 that were uniquely associated with overexpression of the transcription factor *NRRL3_00042*. A search using our database of 968 *Aspergillus*-associated metabolites referenced in literature as well as using the chemical entities of biological importance (ChEBI) database and the MassBank database available within ChemSpider databases did not result in identification of the compounds.

### 3.3. Functional Characterization of the NRPS NRRL3_00036 

**Figure 5:**
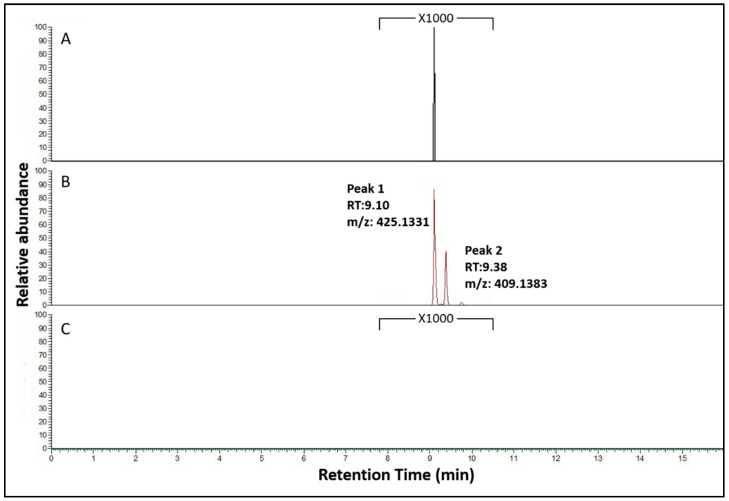
LC-MS analysis of extracts from 6-days-old MM 1% maltose cultures of *A. niger* strains. Extracted ion chromatogram (EIC) of peak 1/compound 1 and peak 2/compound 2. (**A**) EIC of the parent strain expanded 1000 fold; (**B**) EIC of the mutant NRRL3_00042^OE^ strain, (**C**) EIC of the mutant NRRL3_00042^OE^ _ΔNRRL3_00036 strain expanded 1000 fold.

To confirm the role of the NRPS NRRL3_00036 in the production of compounds 1 and 2, we deleted its encoding gene in strain NRRL3_00042^OE^, resulting in the deletion strain NRRL3_00042^OE^_ΔNRRL3_00036. Three independent transformants were isolated, purified and showed the same phenotypic profiles. The deletion of the gene was confirmed by PCR (Figure S3). The phenotype of the deletion mutant strain was similar to the parental strain CSFG_7003 with no pigment in the media observed and growth rate restored (Figure 3). The NRRL3_00042^OE^_ΔNRRL3_00036 strain and the NRRL3_00042^OE^ strain were grown for five days in stationary cultures in maltose inducible conditions. The secreted metabolites were extracted and analyzed by LC-MS. Compounds 1 and 2 overproduced in the strain NRRL3_00042^OE^ were not present in the deletion mutant strain (Figure 4). We expanded the scale by 1000-fold of the region of the chromatograms of the wildtype strain CSFG_7003 and the deletion strain NRRL3_00042^OE^_ΔNRRL3_00036 corresponding to the new compounds produced by NRRL3_00042^OE^, and showed that compound 1 is detectable in the wildtype CSFG_7003 whereas both compounds 1 and 2 are absent in NRRL3_00042^OE^_ΔNRRL3_00036 (Figure 5). These results indicate that the NRPS backbone enzyme gene *NRRL3_00036* is responsible for the production of the compounds 1 and 2 and is under the regulation of the co-localized transcription factor gene *NRRL3_00042*.

### 3.4. Antimicrobial Assays

An antibacterial activity screening was performed on crude extract obtained from the NRRL3_00042^OE^ strain. Growth experiments were done in triplicate and the extracts were tested against the Gram-positive bacterium *Staphylococcus aureus* and the Gram-negative bacterium *Escherichia coli*. There was no evidence of antibacterial activity associated with extracts obtained from the NRRL3_00042^OE^ strain. Bacterial growth proceeded uninhibited in the presence of NRRL3_00042^OE^ crude extracts (Figure S4).

## 4. Discussion

In filamentous fungi, BGCs often include genes encoding a protein predicted to encode fungal-specific transcription factor [20]. Previous studies have shown that overexpression of cluster-linked transcription factor genes is an effective strategy for activating cryptic BGCs and can lead to the production of secondary metabolites [21]. In this study, the phylogenetic and syntenic analysis helped to define the BGC and selecting *NRRL3_00042* as the co-localized transcription factor gene involved in regulation of the BGC. The boundary of the cluster was defined by the common elements of the orthologous clusters. The phylogenetic tree presented in this study has been built using the orthologs of NRRL3_00036 only. We used the taxonomic fungal tree built by the JGI MycoCosm [13] to examine taxonomic distribution of the *NRRL3_00036* cluster. In the Eurotiomycetes, the syntenic *NRRL3_00036* BGC is found only in species in the Aspergilli Nigri and Candidi sections. In the case of *A. cristatus*, the cluster is missing genes encoding the cytochrome P450 and the transporter. The boundary of a BGC provides a convenient reference to describe the genes involved in the biosynthesis of secondary metabolites. However, the biosynthesis of some compounds requires additional unlinked genes. As well, genes located within a BGC may not be required for biosynthesis of secondary metabolites. For example, the biosynthesis of alkylcitrates in *A. niger* requires both clustered and unlinked genes [22]. In another example, the genes involved in the biosynthesis of conidial pigments in *A. fumigatus* [23] and *Alternaria alternate* [24] are clustered in their genomes whereas their orthologs involved in conidial pigment biosynthesis in *A. niger* are unlinked [25]. Moreover, two of the genes in the BGC for conidial pigment biosynthesis in *A. fumigatus*, as defined by co-expression, do not appeared to be involved in conidial pigment biosynthesis [23]. As fungal BGCs evolve rapidly [26], defining the boundary of BGCs and the role of clustered genes in the biosynthesis of secondary metabolites is very challenging and time-consuming [27,28]. Although, in this study, we have defined the *NRRL3_00036* BGC to extend from *NRRL3_00035* to *NRRL3_00043*, we have only provided evidence for the functional involvement of *NRRL3_00036* and *NRRL3_00042* in the production of the two new compounds.

The overexpression of the selected transcription factor confirmed the regulation of the BGC by the NRRL3_00042 transcription factor and resulted in the overproduction of two novel secondary metabolites ~1000 fold higher than the parental strain. The deletion of the gene encoding the NRPS in NRRL3_00042^OE^ restored the wild type phenotype, confirming the role of NRRL3_00036 as backbone enzyme in the production of the novel secondary metabolites in *A. niger*. The two new compounds could not be identified by a search using our internal database of 968 *Aspergillus*-associated metabolites as well as specific chemical databases. Therefore, further work includes the purification of compounds 1 and 2 followed by NMR analysis to resolve the compound structures.

The antibacterial assay was performed against two common human pathogens, the Gram-negative *Escherichia coli* and the Gram-positive *Staphylococcus aureus*. *E. coli* can cause diarrhea and gastroenteritis [29] and *S. aureus* is a major human pathogen that can cause a wide range of diseases [30]. No significant antibacterial activity was detected from the NRRL3_00042^OE^ extract. The Gram-positive *B. subtilis* has been studied for its probiotic properties and is a major industrial host for protein production [31]. *B. subtilis* can grow in co-culture with *A. niger* and it resulted in a down-regulation of this BGC [6]. The antibacterial assay could be extended to *B. subtilis* to test the specificity of the transcriptional response of *A. niger* to *B. subtilis*. In addition, broader activity tests and assays such as antifungal and plant growth factor assay will be considered.

In conclusion, a combinatorial approach of microbial co-cultures, phylogeny, comparative genomics and genome editing led to the characterization of a new biosynthetic gene cluster in *Aspergillus niger* and to the overproduction of novel secondary metabolites.

## Data Availability

Not applicable.

## Supplementary Materials

The following are available online at https://www.mdpi.com/article/10.3390/jof7050374/s1, Table S1. Primers and oligonucleotides used in this study. Table S2. *Aspergillus niger* strains. Figure S1. Verification of NRRL3_00042 over-expression strain. Figure S2. Verification of *NRRL3_00042* and *NRRL3_00036* expression in NRRL3_00042^OE^ and CSFG_7003 by RT-PCR. Figure S3. Verification of NRRL3_00036 deletion strain. Figure S4. *Escherichia coli* JW5503–1 and *Staphylococcus aureus* N315 inhibition curves.
