# Supplementary material for: Identification of a Novel Biosynthetic Gene Cluster in Aspergillus niger Using Comparative Genomics
Source: J Fungi (Basel). 2021 May 11;7(5):374. doi: 10.3390/jof7050374 (PMC8151901; doi:10.3390/jof7050374)
Supplement: Supplementary file 1 [file jof-07-00374-s001.zip › jof-1190437-supplementary.pdf]

Supplementary Materials

Identification of a Novel Biosynthetic Gene Cluster in *Aspergillus niger* Using Comparative Genomics

[1] Centre for Structural and Functional Genomics, Department of Biology, Concordia University, 7141 Rue Sherbrooke Ouest, Montréal, Québec H4B 1R6, Canada; gregory.evdokias@gmail.com (G.E.); montse.mora.ochomogo@gmail.com (M. M.-O.); marcos.difalco@concordia.ca (M.D.F.); thitrucminh.nguyen@concordia.ca (T.T.M.N.); adrian.tsang@concordia.ca (A.T.)
[2] Department of Microbiology, Immunology and Infectious Disease, University of Calgary, 3330 Hospital Drive, Calgary, Alberta T2N 4N1, Canada; cjsemper@ucalgary.ca (C.S.); alexei.savchenko@ucalgary.ca (A.S.)
* Correspondence: isabelle.benoit@concordia.ca

Table S1. Primers and oligonucleotides udes in this study.

Name	Nucleotide Sequences (5'>3')	Description
NRRL3_00042OE_FW	TACTTCCAATCCAATCCATTTTTA-GAT**ATGAG-TCCCGTGTCTGGCCAG**	Forward primer for PCR amplification of *NRRL3_00042* and insertion at the *gla*A locus
NRRL3_00042OE_RV	TTATCCAC-TTCCAATCCATTTTTAG-**TTACATATTCCATGCAAAC-GATCCATCTTC**	Reverse primer for PCR amplification of *NRRL3_00042* and insertion at the *gla*A locus
*gla*A_gRNA	CGACGGTGACTGACACCTGG	Guide RNA targeting *gla*A.
*glaA*_replacement_Chk_Fw	CCAGCATCATTACACCTCAGCA	Forward primer for PCR verification of *NRRL3_00042* insertion behind the glucoamylase promoter.
*glaA*_replacement_Chk_Rv	CCTTTAAC-TATAGCGAAATGGATTGATTGT	Reverse primer for PCR verification of *NRRL3_00042* insertion behind the glucoamylase promoter.
NRRL3_00036_gRNA	GTACACCCGCAACTTTACCT	Guide RNA targeting *NRRL3_00036*.
NRRL3_00036_RO	*TAGCGGGTCTCAATTCCG-CAGGCACATCTCAGCTCG-CATGTCGAC*CATCAAACCGGAC-CATCCCCAATGCAGTGTCTAAGCAACATCCCG	Rescue oligonucleotide for *NRRL3_00036* deletion.
NRRL3_36KO_Chk_Fw	GTGGATGTCCAAGCAACCAC	Forward primer for PCR verification of NRRL3_00036 deletion.
NRRL3_36KO_Chk_Rv1	GTGATAGCACGACCGTTGATG	Reverse primer for PCR verification of NRRL3_00036 deletion.
NRRL3_36KO_Chk_Rv2	GACCAAGCATTATCAC-GGAGGTTC	Reverse primer for PCR verification of *NRRL3_00036* deletion.
NRRL3_00036_RTPCR_Fw	TCCTGGGTGAAGTGGTCCGTC	Forward primer for RT-PCR verification of *NRRL3_00036* expression.
NRRL3_00036_RTPCR_Rv	CAACTTCTCAACACTCTGGATCC	Reverse primer for RT-PCR verification of *NRRL3_00036* expression.
NRRL3_00042_RTPCR_Fw	GGACCTGCAGACCTCTATCG	Forward primer for RT-PCR verification of *NRRL3_00042* expression.
NRRL3_00042_RTPCR_Rv	CTGTGTGGTTTGGAG*CATTT-GTGC	Reverse primer for RT-PCR verification of *NRRL3_00042* expression.
Aniger_Btubulin_Fw	ACAACTGCCATTGGATTGGG	Forward primer for RT-PCR -tubulin positive control.

Aniger_Btubulin_Rv	GTTGTTACCAGCACCGGACT	Reverse primer for RT-PCR -tubulin positive control.

Note: The beginning of the gene *NRRL3_00042* is in **bold**; the promotor and terminator regions of the glucoamylase gene (glaA) are underlined; forward 5′-3′ sequence of the rescue oligonucleotide (RO) for *NRRL3_00036* is in *italic.* NRRL3_00042_RTPCR_Rv has been designed to span over an intron allowing to anneal only to cDNA and not to gDNA, the * indicates the junction between the two exons in *NRRL3_00042*. *NRRL3_00036* does not have an intron.

Table S2. *Aspergillus niger* strains.

**CSFG_7003**	**NRRL2270 ΔpyrG ΔkusA**	**This study**
NRRL3_00042^{OE}	NRRL2270 *Δ*kusA glaA::NRRL3_00042	This study
NRRL3_00036^{KO}	NRRL2270 ΔkusA glaA::NRRL3_00042 ΔNRRL3_00036	This study

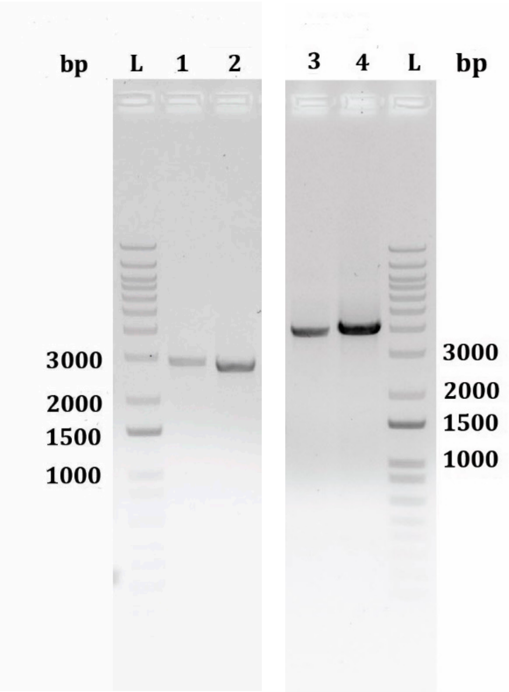

Figure S1. Verification of *NRRL3_00042* over-expression strain. PCR amplification and corresponding restriction enzyme digestion were ran on electrophoresis agarose gels. Lanes 1 and 2 are the NRRL3_00042^{OE} strain, lanes 3 and 4 are the parental strain CSFG_7003. L: molecular size ladder; Lane 1: PCR amplification of the gene NRRL3_00042 with primers glaA_replacement_Chk_FW and glaA_replacement_Chk_RV from genomic DNA from the NRRL3_00042OE strain. The PCR product is at the expected size of 2721 bp.; Lane 2: PCR product from lane 1 was digested by the restriction enzyme BglII. The digestion products are expected at 2601 and 120 bp. The band at 120 bp is faint but visible when the contrast is increased; Lane 3: PCR amplification of the gene encoding glycoamylase with primers glaA_replacement_Chk_FW and glaA_replacement_Chk_RV from genomic DNA of the parental strain. The PCR product is at the expected size of 3658 bp.; Lane 4: PCR product from lane 1 was digested by the restriction enzyme BglII. As there is no BglII restriction site in the glucoamylase gene, the corresponding digestion product expected is 3658 bp.

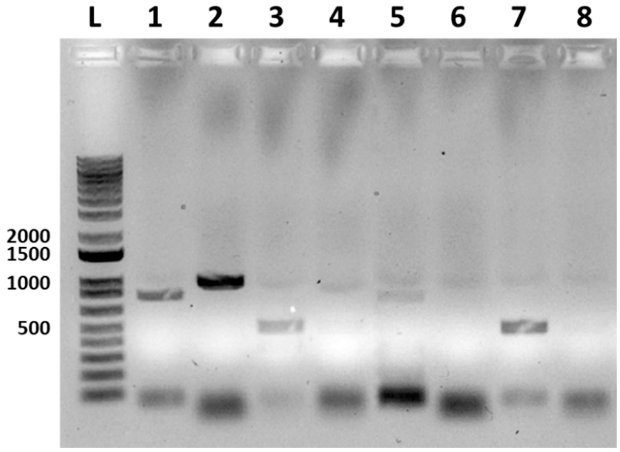

Figure S2. Verification of the expression of the *NRRL3_00036* and *NRRL3_00042* genes in the NRRL3_00042^{OE} strain and the parental strain CSFG_7003 by reverse transcription PCR. RT-PCR products were run on electrophoresis agarose gels; L: Molecular size ladder (bp).; Lane 1: Complementary DNA from the NRRL3_00042OE strain, RT-PCR amplification using the primers NRRL3_00036_RTPCR_Fw and NRRL3_00036_RTPCR_Rv. The PCR product is at the expected size of 794 bp.; Lane 2: Complementary DNA from the NRRL3_00042OE strain, RT-PCR amplification using primers NRRL3_00042_RTPCR_Fw and NRRL3_00042_RTPCR_Rv. The PCR product is at the expected size of 991 bp. NRRL3_00042_RTPCR_Rv has been designed to span over an intron allowing to anneal only to cDNA and not to gDNA.; Lane 3: Complementary DNA from the NRRL3_00042OE strain, RT-PCR amplification using primers BTubulin_Fw and BTubulin_Rv. The PCR product is at the expected size of 500 bp.; Lane 4: Negative control NRT. Complementary DNA from the NRRL3_00042OE strain, RT-PCR amplification using primers for NRRL3_00036 and NRRL3_00042 (multiplex) and no reverse transcriptase to assess possible genomic DNA contamination; Lane 5: Complementary DNA from the CSFG_7003 strain, RT-PCR amplification using the primers NRRL3_00036_RTPCR_Fw and NRRL3_00036_RTPCR_Rv; Lane 6: Complementary DNA from the CSFG_7003 strain, RT-PCR amplification using primers NRRL3_00042_RTPCR_Fw and NRRL3_00042_RTPCR_Rv; Lane 7: Complementary DNA from the CSFG_7003 strain, RT-PCR amplification using primers BTubulin_Fw and BTubulin_Rv. The PCR product is at the expected size of 500 bp.; Lane 8: Negative control NRT. Complementary DNA from the CSFG_7003 strain, RT-PCR amplification using primers for NRRL3_00036 and NRRL3_00042 (multiplex) and no reverse transcriptase to assess possible genomic DNA contamination.

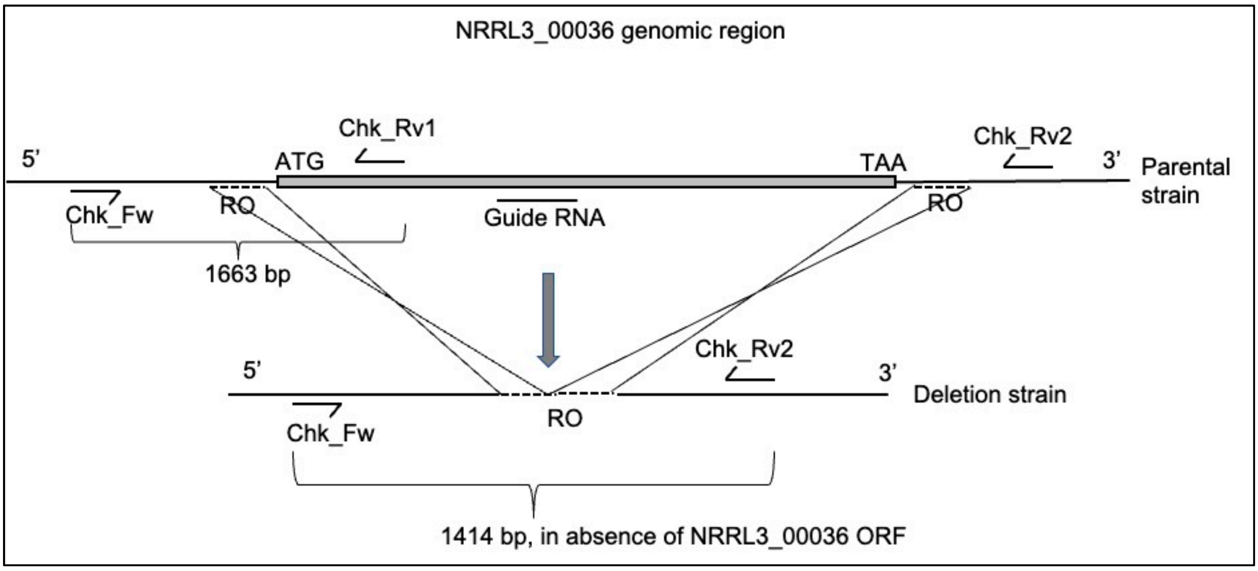

Note: RO, rescue oligonucleotide.

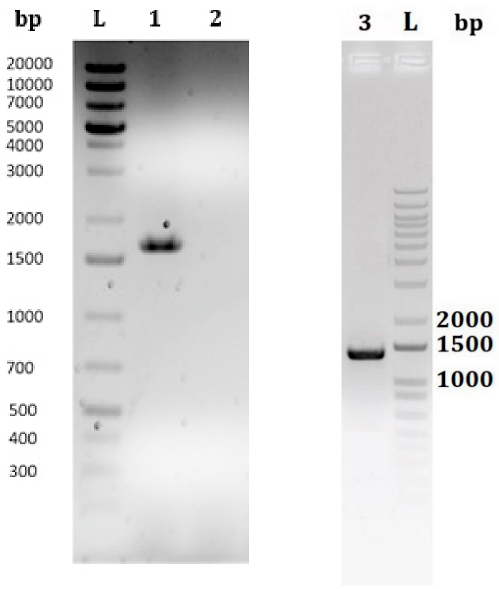

Figure S3. Verification of *NRRL3_00036* deletion strain. Amplification of the *NRRL3_00036* locus in NRRL3_00042^{OE} strain and NRRL3_00036 strain. PCR products were run on electrophoresis agarose gels; L: molecular size ladder; Lane 1: PCR amplification of the gene NRRL3_00036 with primers NRRL3_36KO_Chk_Fw and NRRL3_36KO_Chk_Rv1 from genomic DNA of the NRRL3_00042OE strain. The PCR product is at the expected size of 1663 pb.; Lane 2: PCR amplification of the gene NRRL3_00036 with primers NRRL3_36KO_Chk_Fw and NRRL3_36KO_Chk_Rv1 from genomic DNA of the NRRL3_00036 strain. No PCR product expected in the absence of NRRL3_00036; Lane 3: PCR amplification of the gene NRRL3_00036 locus with primers NRRL3_36KO_Chk_Fw and NRRL3_36KO_Chk_Rv2 from genomic DNA of the NRRL3_00036 strain. The PCR product in the absence of NRRL3_00036 is at the expected size of 1414 bp.

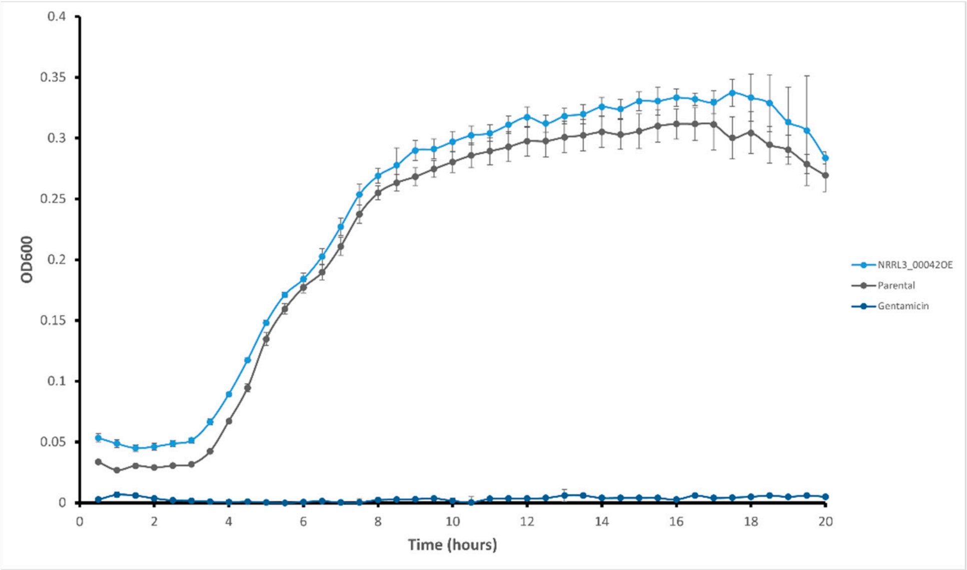

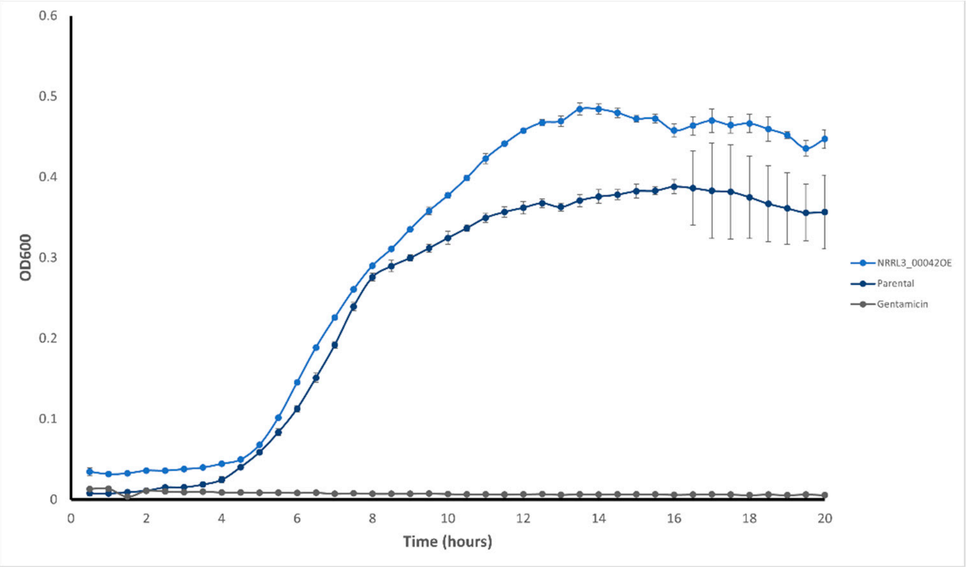

Figure S4. *Escherichia coli* JW5503-1 and *Staphylococcus aureus* N315 inhibition curves. The antibiotic gentamicin is the positive control, the parental strain is CSFG_7003, the NRRL3_00042OE is CSFG_7003 overexpressing the gene NRRL3_00042. Error bars show the average of biological triplicates.
